# Supplementary material for: Human pluripotent reprogramming with CRISPR activators
Source: Nat Commun. 2018 Jul 6;9:2643. doi: 10.1038/s41467-018-05067-x (PMC6035213; doi:10.1038/s41467-018-05067-x)
Supplement: Supplementary file 1 — Supplementary Information [file 41467_2018_5067_MOESM1_ESM.pdf]

Supplementary Information

Human pluripotent reprogramming with CRISPR activators

Weltner et al.

Supplementary Note 1 | P300 core containing activators

We tested additional dCas9 activator constructs that contained an extra P300 core domain after the VP192 domain for their use in cellular reprogramming. These activators contain either VP192-P300 domains (VPP300) or VP192-P300-P65-HSF1 domains (VPPH) (Supplementary Fig. 4a). The gene activation pattern with VPP300 and VPPH using different single guide RNAs in transiently transfected HEK293 cells was very similar to that seen with dCas9VPH (Fig. 2b and Supplementary Fig. 4b).

The efficiency of the different activators was compared in selected HEK293 cells expressing destabilized DDdCas9 activators that can be induced by trimethoprim addition, and OMKLS gRNA plasmid (Fig. 2b). Activation of *OCT4* was most efficient with dCas9VPP300 activator at both day 1 and day 3 time points (Supplementary Fig. 4c). Other genes did not show consistent and statistically significant difference in activation efficiency in both day 1 and day 3 time points, although *SOX2* activation was improved at day 1 time point by VPP300 (Supplementary Fig. 4c). *LIN28* and *OCT4* expression had an increasing trend between days 1 and 3, particularly with dCas9VPP300 activation, suggesting that the activation of these genes may take longer than the other ones and be more amenable to improvement by targeted histone acetylation. The activation of *SOX2* and *KLF4* peaked at day 1 samples with P300 core containing activators, whereas VP192 and VPH mediated activation of these genes had an increasing trend between days 1 and 3. These genes are already expressed at relatively high levels in HEK293 cells, which may affect their expression dynamics during targeted activation. MYC activation was weaker in P300 core containing conditions and peaked in all conditions at day 1. It is possible that the transcriptional activation of the other reprogramming factors contributes to the feed-back inhibition of the endogenous MYC levels. These results suggest that P300 core containing activators, particularly VPP300, may promote more efficient transient activation, but they may also have gene-dependent and temporally affected effects.

The reprograming efficiency with dCas9VPP300 and dCas9VPPH in episomal plasmid based CRISPRa reprogramming is lower than with the dCas9VP192 activator (Supplementary Fig. 4c and Fig. 3g). Reprogramming with dCas9VPP300 and dCas9VPPH also show reduced efficiency when targeting *OCT4* (Supplementary Fig. 3b), but not in fully transgenic transcription factor mediated reprogramming (Supplementary Fig. 3c). This may be caused by negative on-target effects that may affect persistence of *OCT4* expression (e.g. sterical impediment effects or interfering acetylation), excessive OCT4 expression resulting in imbalanced reprogramming transcription factor stoichiometry or the size of the plasmids impacting their delivery efficiency and replicative maintenance.

It is also possible that the P300 core containing factors may have a negative off-target effects on the cells when the activator proteins are expressed in high levels. Staining of HEK293 transfected with the different activators for acetylated Histone 3 demonstrated subpopulation of cells with increased nuclear H3 acetylation (Supplementary Fig. 4e). The number of high H3 acetylated cells was increased by the presence of the P300 core domain, as seen by an elongated tail in the FACS histograms (Supplementary Fig. 4f). This increase in global H3 acetylation may end up affecting the final gene activation and reprogramming efficiency. It may also be worthy to further investigate the effect of different activator domains in various vector systems or guide combinations, which may provide more optimal expression levels or reprogramming kinetics.

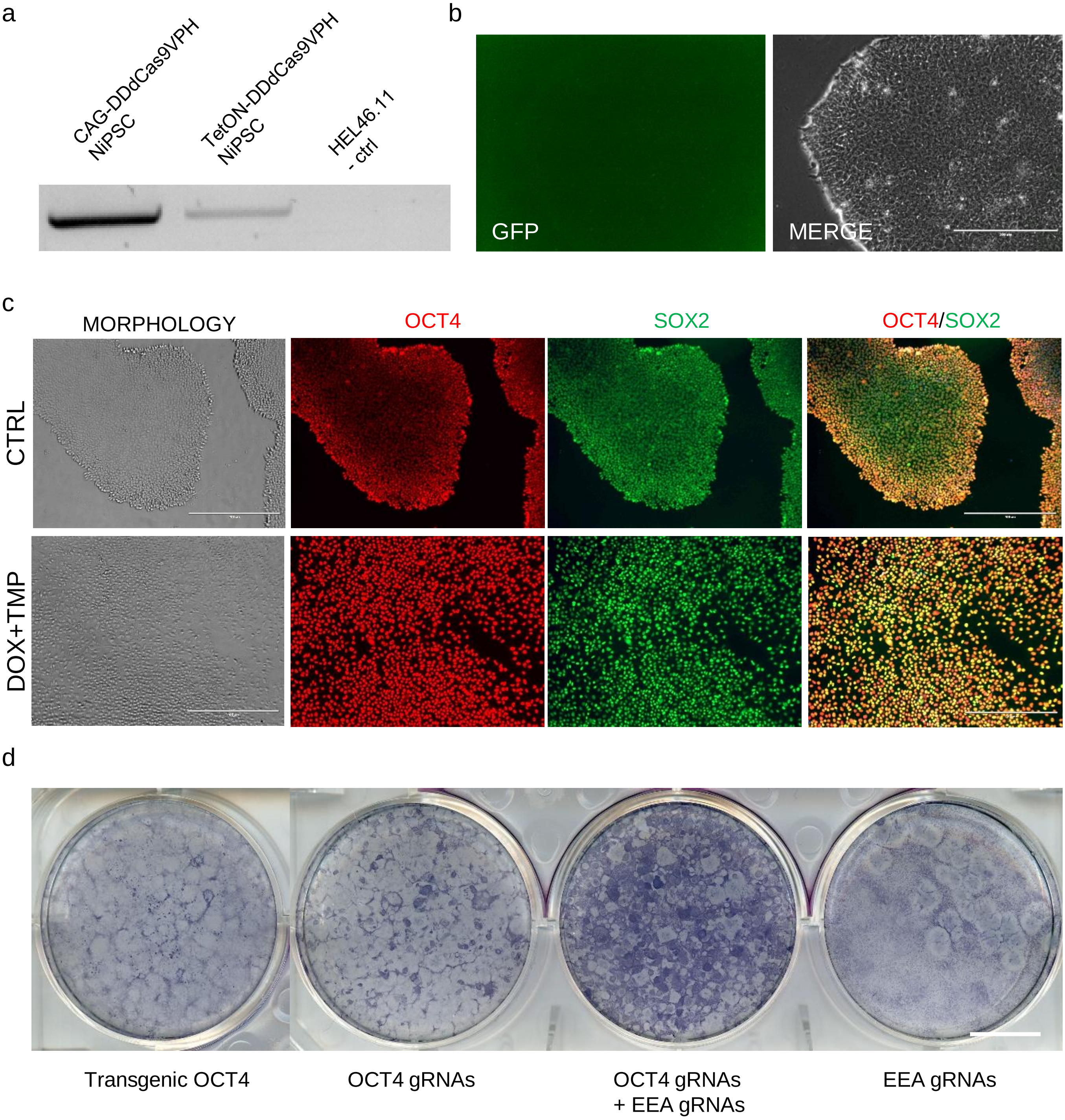

Supplementary Figure 1 | Characterization of NSC derived CRISPR iPSC (NiPSC), related to Figure 1. (**a**) Genomic DNA PCR showing the presence of dCas9 amplicon in the NiPSC. (**b**) Absence of GFP expression (DDdCas9VPH-T2A-GFP) in the NiPSC colonies. Scale bar 200µm. (**c**) Disruption of colony morphology of CRISPR NiPSCs upon *OCT4* re-targeting by doxycycline and trimethoprim treatment. Scale bar 400 µm. (**d**) Alkaline phosphatase staining of NSC induced by CRISPRa-mediated *OCT4* targeting, EEA-motif targeting, and transgenic OCT4. Scale bar 10 mm.

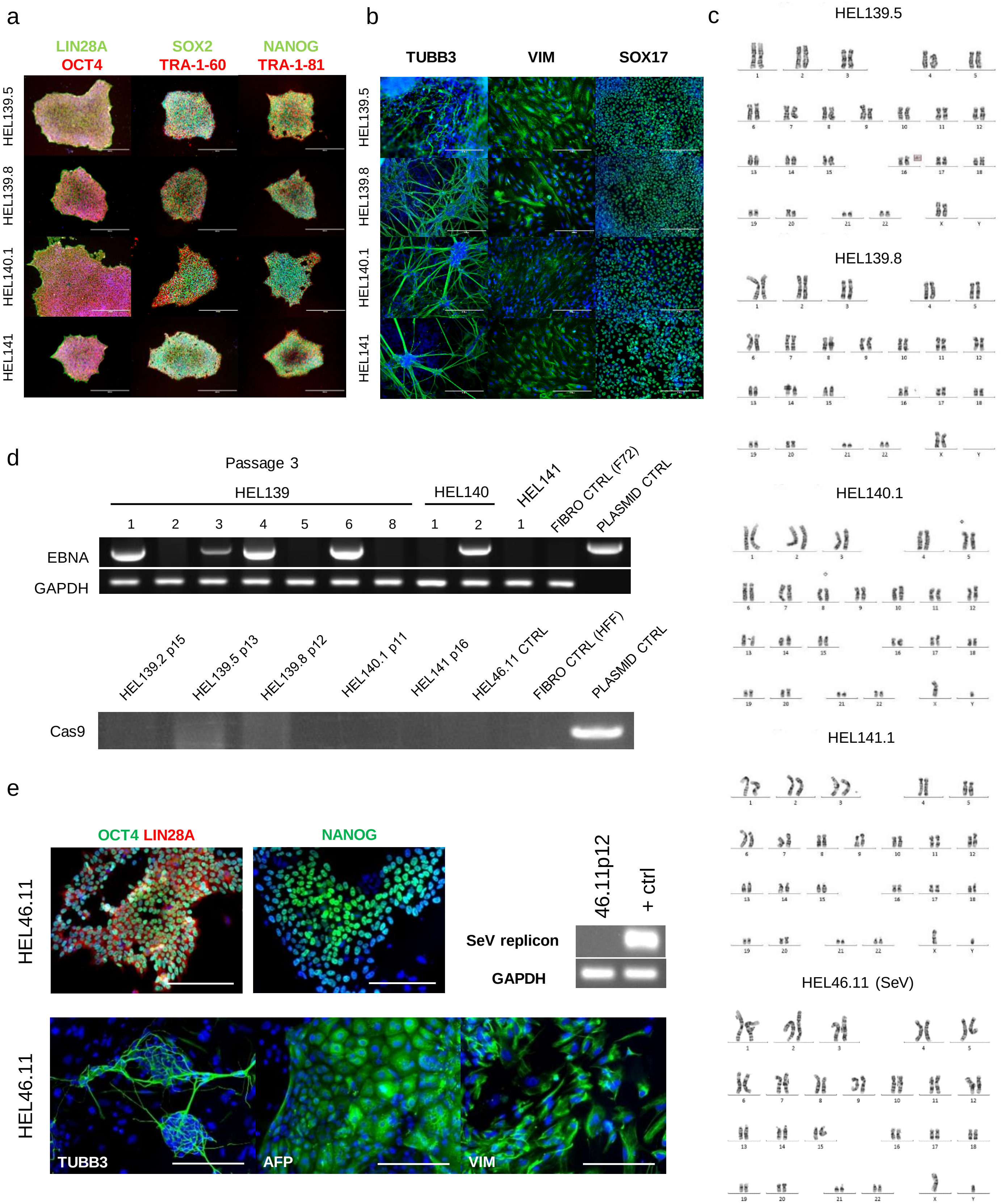

Supplementary Figure 2 | Pluripotency characterization of iPCS lines, related to Figure 3. (**a**) Episomal plasmid derived CRISPR iPSCs from HFFs (HEL140, 141) and F72 fibroblasts (HEL139) are positive for pluripotency associated factors. Scale bar 400 µm (**b**) Embryoid body differentiation of the cell lines into three embryonic germ layers: ectoderm (TUBB3), mesoderm (VIMENTIN) and endoderm (SOX17) (green). Scale bar 200 µm. (**c**) Karyotypes of HEL139.5 (46, XX), HEL139.8 (46, XX), HEL140.1 (46, XY, 75% of analysed cells), HEL141 (46, XY, abn(3q)) and SeV control line HEL46.11 (46, XX). (**d**) Episome detection by PCR with EBNA primers at passage 3 and Cas9 primers at later passages of CRISPR dCas9VPH reprogrammed iPSCs. (**e**) Pluripotency, differentiation and absence of viral episome characterization of control SeV iPSC line HEL46.11. Scale bar 200 µm. Nuclei stained blue.

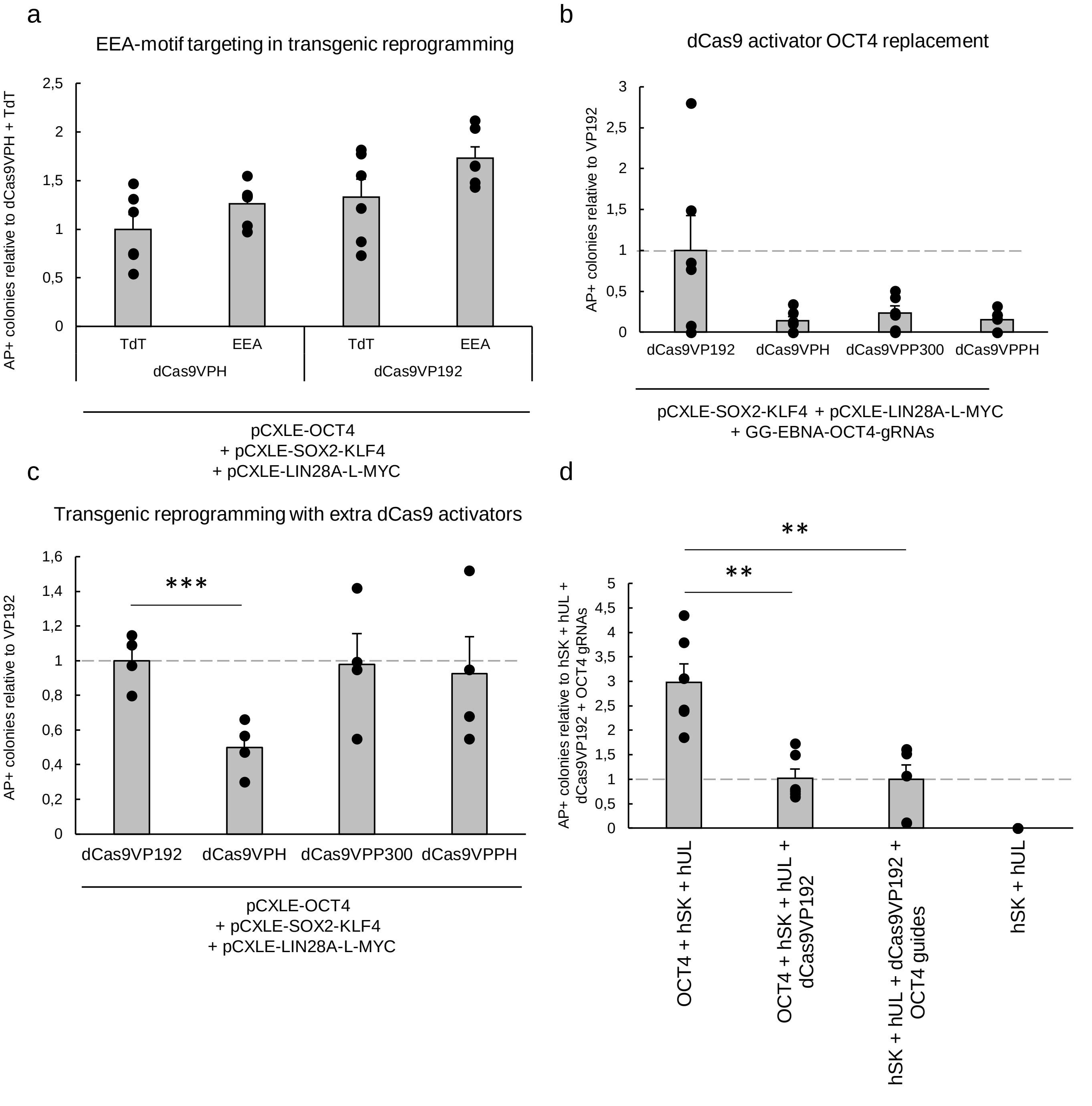

Supplementary Figure 3 | Effect of dCas9 activators on reprogramming efficiency, related to Figure 3. (**a**) The effect of EEA-gRNAs on transgenic reprogramming of HFF with OCT4, SOX2, KLF4, LIN28 and L-MYC. n = 6, 3 independent experiments. (**b**) Reprogramming of HFF with transgenic SOX2, KLF4, L-MYC and LIN28A with dCas9 activator mediated *OCT4* targeting. n = 6, 3 independent experiments. (**c**) Reprogramming of HFF with transgenic OCT4, KLF4, L-MYC and LIN28A with extra dCas9 activator plasmid without gRNAs. n = 4, 3 independent experiments. (**d**) Control inductions for b and c. n = 6, 3 independent experiments. Data presented as mean ± s.e.m., two tailed Student's t-test. ** P<0.01, *** P<0.001. hSK = pCXLE-SOX2-KLF4, hUL = pCXLE-LIN28A-L-MYC

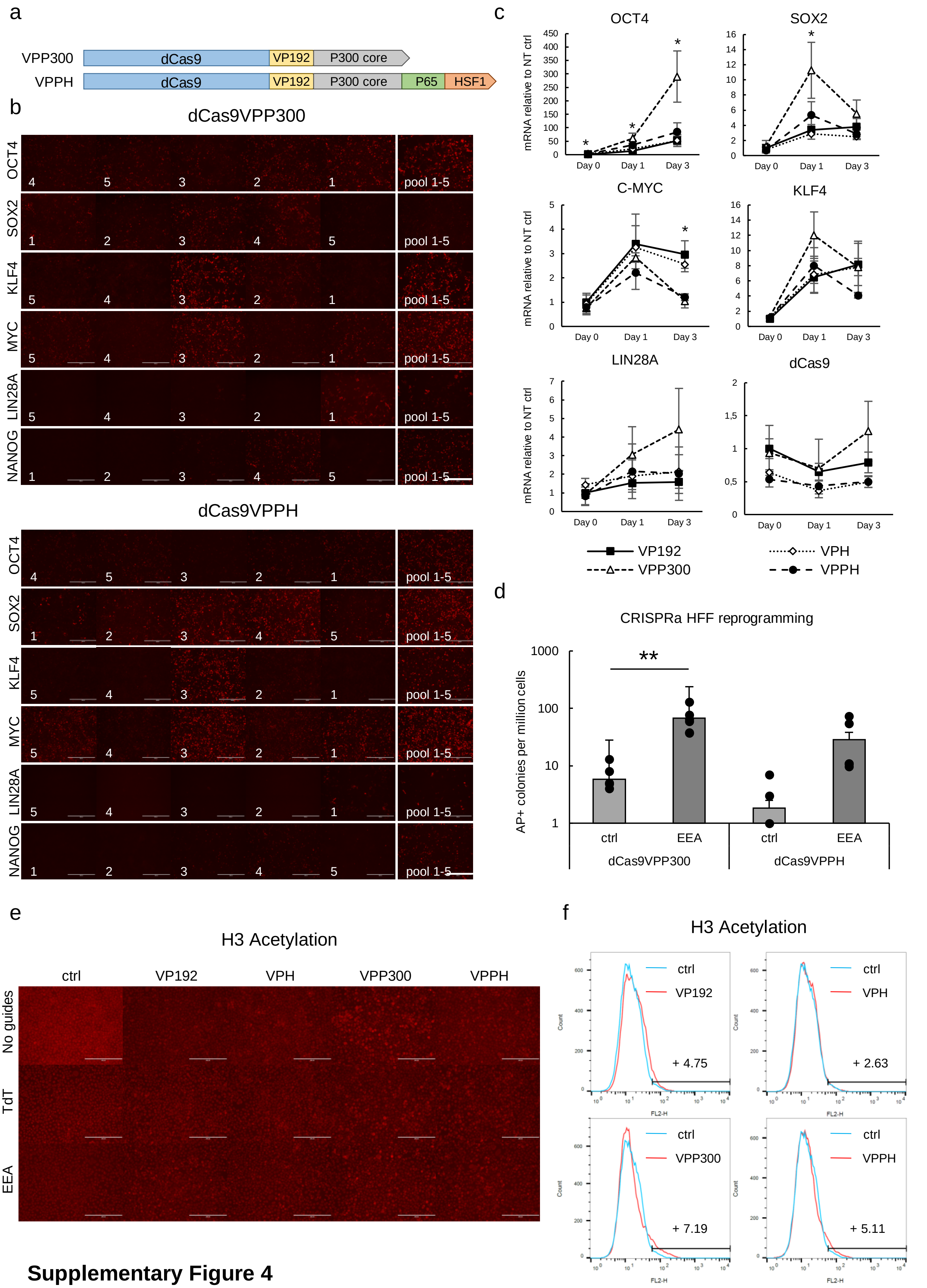

Supplementary Figure 4

Supplementary Figure 4 | Gene activation and reprogramming with P300 core fusion activators, related to Figures 2 and 3 and Supplementary note. (**a**) Schematic representation of different P300 core fused dCas9 activator constructs tested. (**b**) Immunocytochemical staining of reprogramming factors after single gRNA activation and pooled mixture of five guides in HEK293 with dCas9VPP300 and dCas9VPPH. Scale bar 400 µm. (**c**) Reprogramming factor activation, using constitutively expressed DDdCas9 effectors with different activation domains, in HEK293 by qPCR after TMP addition. n = 3, data are from 3 independent experiments. Error bars represent s. d. One way Anova with Tukey HSD test used for statistical comparisons. (**d**) Effect of different P300 core fused dCas9 activator constructs and EEA-motif targeting on CRISPRa reprogramming efficiency of HFFs. n = 6 from 3 independent experiments. Data presented as mean ± s.e.m., two tailed Student's t-test. ** P<0.01 (**e**) Immunocytochemical staining of acetylated Histone 3 tails in HEK293 transfected with different dCas9 activators constructs and EEA-gRNAs, TdTomato control guide (TdT-g1) or no guides. Scale bar 200 µm (**f**) FACS quantification of H3 acetylation bright cells in TdT-g1 and dCas9 activator transfected HEK293 cells.

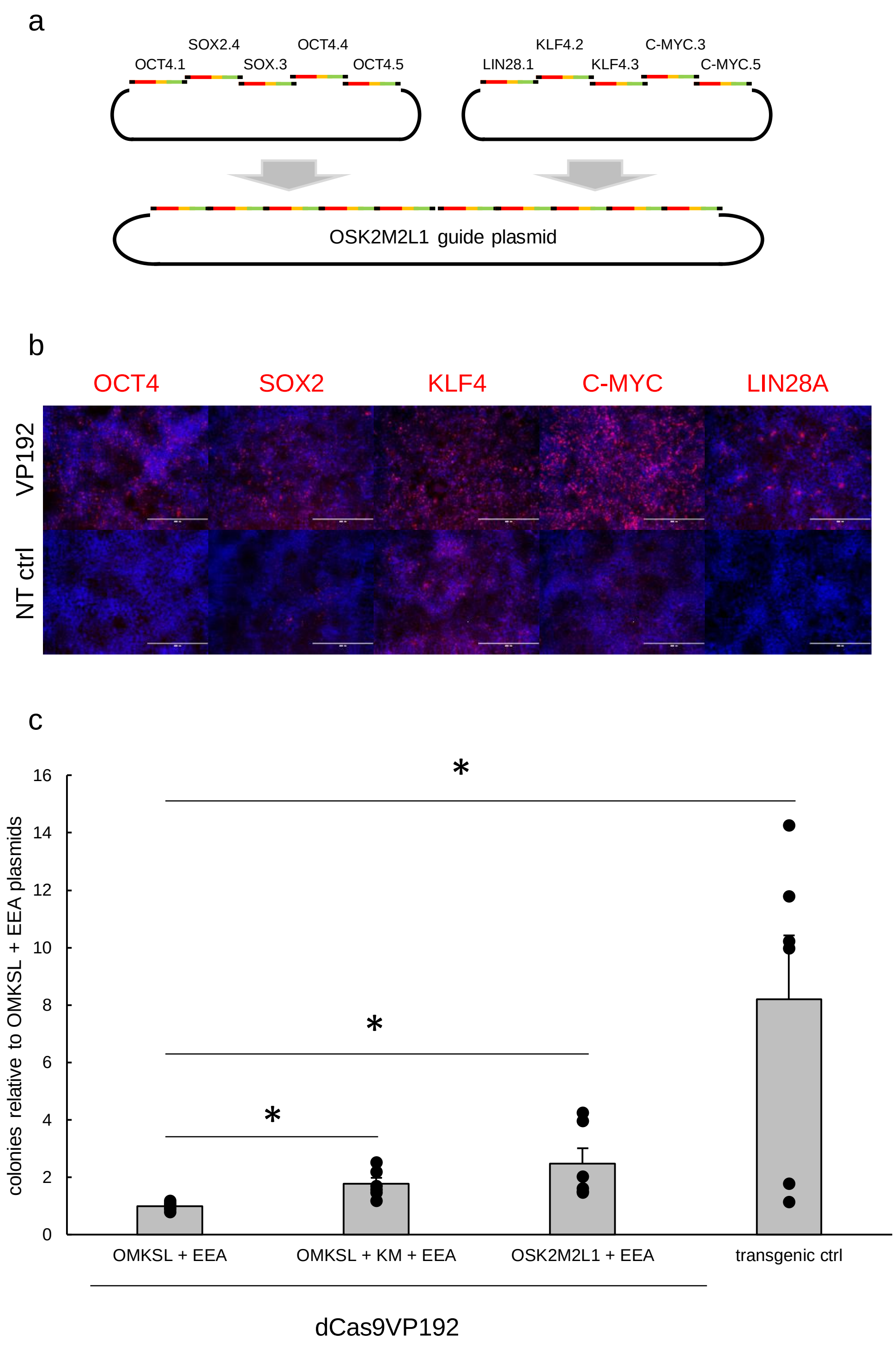

Supplementary Figure 5 | Core reprogramming factor guide optimization for single plasmid delivery, related to Figure 5. (**a**) Schematic representation of concatenated core guide plasmid OSK2M2L1 construction. Notice different number of LIN28A, KLF4 and C-MYC guides compared to OMKSL plasmid (Fig. 2) (**b**) Validation of target gene activation with core factor plasmid in HEK293. Cells transfected with the concatenated reprogramming factor guide plasmid and dCas9-VP192 plasmid, and non-transfected controls (NT). Scale bar 400 µm. Nuclei stained blue. (**c**) Reprogramming efficiencies of HFFs relative to OMKSL and EEA-motif gRNA plasmid. KM plasmid contains five gRNAs targeting KLF4 and five gRNAs targeting MYC. Error bars represent s.e.m., n = 6, 3 independent experiments. Data presented as mean ± SEM, two tailed Student's t-test. * P<0.05

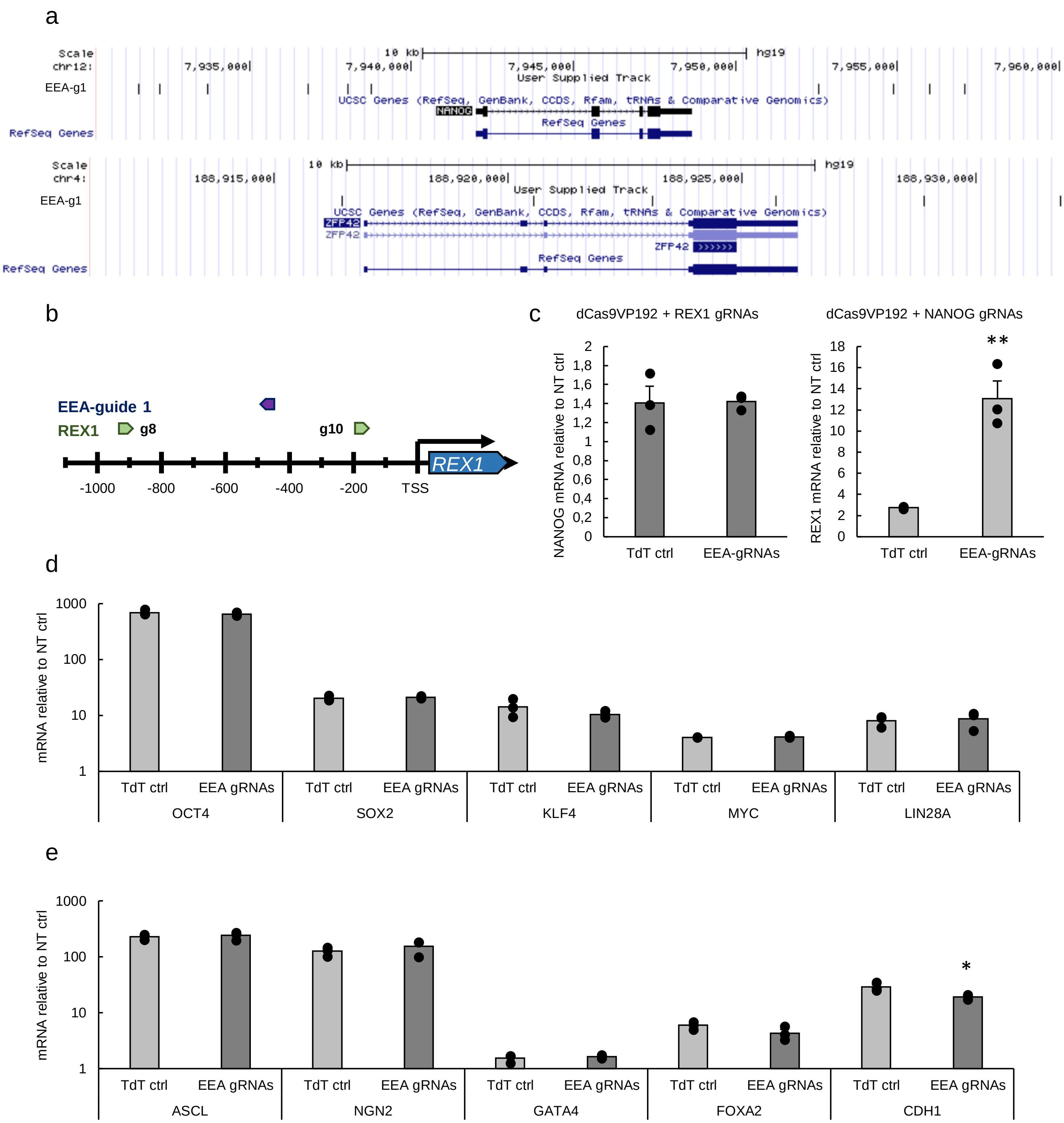

Supplementary Figure 6 | EEA-motif targeting does not improve activation of all pluripotent reprogramming factors, related to Figure 5. (**a**) Positions of EEA-gRNA 1 sites near *NANOG* and *REX1* genes. (**b**) Schematic representation of *REX1* promoter targeting showing *REX1* guide positions and a position of EEA-guide1 between the *REX1* activation guides. (**c**) Simultaneous targeting of *REX1* and EEA-motif does not affect *NANOG* activation, whereas simultaneous targeting of EEA-motif with *NANOG* gRNAs increases *REX1* activation in an EEA-motif guide dependent manner. HEK293 cells transiently transfected with dCas9VP192 and gRNA plasmids. (**d** and **e**) Simultaneous EEA-motif targeting does not have consistent effect in improving activation of *OCT4*, *SOX2*, *KLF4*, *LIN28A* or *MYC* reprogramming factors (**d**), or other factors *ASCL1*, *NGN2*, *GATA4*, *FOXA2* or *CDH1* (**e**). HEK293 cells transiently transfected with dCas9VP192 and gRNA plasmids. n = 3. Data presented as mean ± s.e.m., two tailed Student's t-test. * P<0.05, ** P<0.01

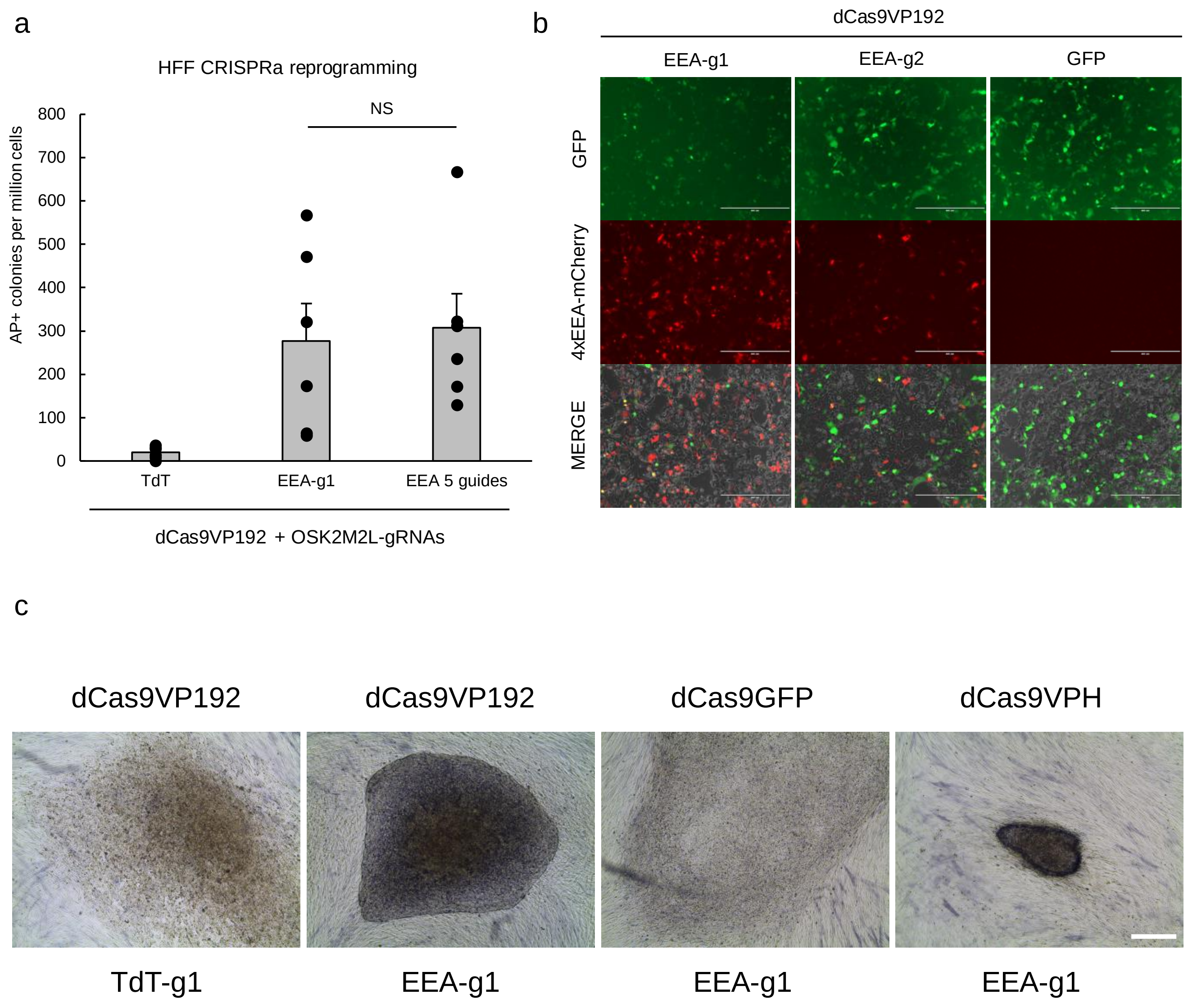

Supplementary Figure 7 | EEA-motif targeting by EEA-g1, related to Figure 6. (**a**) Effect of EEA-g1 and 5 guides targeting the EEA-motif in CRISPRa reprogramming of HFFs using dCas9VP192 and OSK2M2L1 gRNAs. Error bars represent SEM, n = 6, 3 independent experiments. Data presented as mean ± s.e.m., two tailed Student's t-test. (**b**) mCherry reporter activation with EEA-motif gRNAs 1 and 2, and pXMs-DD-GFP control in reporter transfected HEK293. Scale bar 400 µm. (**c**) Alkaline phosphatase stained colonies induced by transgenic OCT4, SOX2, LIN28A and L-MYC in the presence of different dCas9 effectors and EEA-g1. Only dCas9 activators with EEA-g1 induce iPSC-like alkaline phosphatase positive colonies. Scale bar 400 µm.

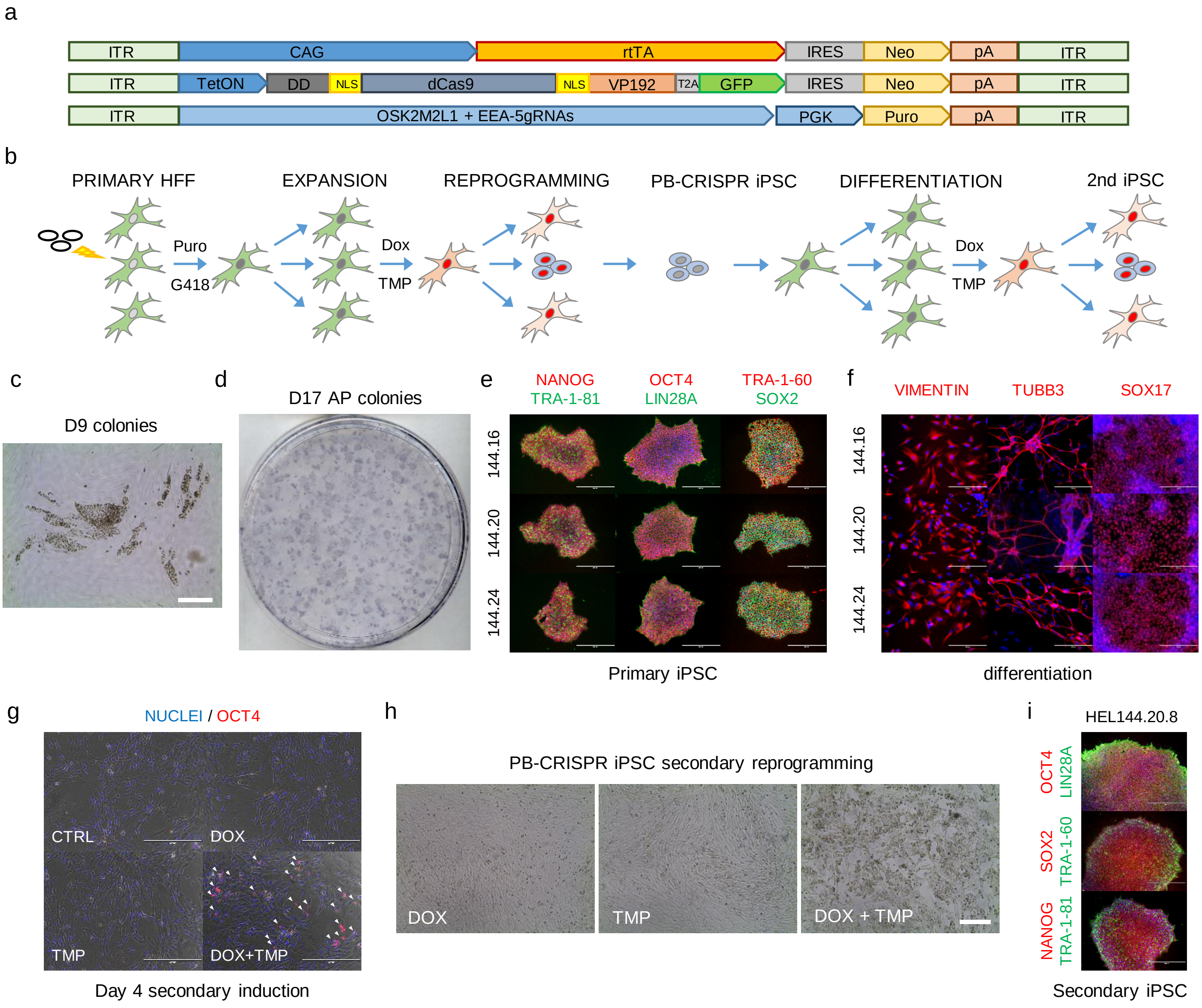

Supplementary Figure 8 | Characterization of PiggyBac CRISPRa iPSC reprogramming. (**a**) Schematic representation of vectors used in PiggyBac reprogramming. (**b**) Schematic representation of the reprogramming process. (**c**) Emerging colonies from primary HFF iPSC induction at day 9 of reprogramming in the presence of doxycycline (DOX) and trimethoprim (TMP). Scale bar 400 µm. (**d**) Alkaline phosphatase positive colonies from primary HFF cells at day 17 of reprogramming. (**e**) Pluripotency marker expression in PB-CRISPRa iPSCs derived from primary HFFs. Scale bar 400 µm. (**f**) Embryoid body differentiation of PB-CRISPRa iPSC into three embryonic germ layer derivatives in vitro. Scale bar 200 µm. (**g**) DOX and TMP dependent activation of CRISPRa targeted OCT4 (red) at day 4 of secondary reprogramming from differentiated fibroblast-like cells derived from HEL144.20. Scale bar 400 µm. (**h**) DOX and TMP dependent morphological changes in fibroblast-like cells differentiated from PB-CRISPR iPSCs. Scale bar 400 µm. (**i**) Pluripotency marker expression in secondary PB-CRISPRa iPSCs at passage 3. Scale bar 400µm. Nuclei stained blue.

Supplementary Table 1 | Guide RNA oligos, related to guide RNA design and production method

	gene	guide nr	oligo sequence
1	OCT4	1	GTGGAAAGGACGAAACACCG GGGGGAGAAACTGAGGCGA GTTTTAGAGCTAGAAATAG
2	OCT4	2	GTGGAAAGGACGAAACACCG GGTGGTGGCAATGGTGTCTG GTTTTAGAGCTAGAAATAG
3	OCT4	3	GTGGAAAGGACGAAACACCG GACACAACTGGCGCCCCTCC GTTTTAGAGCTAGAAATAG
4	OCT4	4	GTGGAAAGGACGAAACACCG GGCACAGTGCCAGAGGTCTG GTTTTAGAGCTAGAAATAG
5	OCT4	5	GTGGAAAGGACGAAACACCG TCTGTGGGGGACCTGCACTG GTTTTAGAGCTAGAAATAG
6	SOX2	1	GTGGAAAGGACGAAACACCG TGTAAGGTAAGAGAGGAGAG GTTTTAGAGCTAGAAATAG
7	SOX2	2	GTGGAAAGGACGAAACACCG TTTACCCACTTCCTTCGAAA GTTTTAGAGCTAGAAATAG
8	SOX2	3	GTGGAAAGGACGAAACACCG GTGGCTGGCAGGCTGGCTCT GTTTTAGAGCTAGAAATAG
9	SOX2	4	GTGGAAAGGACGAAACACCG CAAAACCCGGCAGCGAGGCT GTTTTAGAGCTAGAAATAG
10	SOX2	5	GTGGAAAGGACGAAACACCG AGGAGCCGCCGCGCGCTGAT GTTTTAGAGCTAGAAATAG
11	KLF4	1	GTGGAAAGGACGAAACACCG CGAACGTGTCTGCGGGCGCG GTTTTAGAGCTAGAAATAG
12	KLF4	2	GTGGAAAGGACGAAACACCG TATAAGTAAGGAACGCGCGC GTTTTAGAGCTAGAAATAG
13	KLF4	3	GTGGAAAGGACGAAACACCG GCTGCCATAGCAACGATGGA GTTTTAGAGCTAGAAATAG
14	KLF4	4	GTGGAAAGGACGAAACACCG GTTCGGTCGCTGCGCGACCA GTTTTAGAGCTAGAAATAG
15	KLF4	5	GTGGAAAGGACGAAACACCG TCTTCGCGGGCTTCGAACCC GTTTTAGAGCTAGAAATAG
16	MYC	1	GTGGAAAGGACGAAACACCG CCCTTTATAATGCGAGGGTC GTTTTAGAGCTAGAAATAG
17	MYC	2	GTGGAAAGGACGAAACACCG TCTCGCTAATCTCCGCCCAC GTTTTAGAGCTAGAAATAG
18	MYC	3	GTGGAAAGGACGAAACACCG GGTTCCCAAAGCAGAGGGCG GTTTTAGAGCTAGAAATAG
19	MYC	4	GTGGAAAGGACGAAACACCG AGCTAGAGTGCTCGGCTGCC GTTTTAGAGCTAGAAATAG
20	MYC	5	GTGGAAAGGACGAAACACCG GCGCGCGTAGTTAATTCATG GTTTTAGAGCTAGAAATAG
21	LIN28A	1	GTGGAAAGGACGAAACACCG GTGTCAGAGACCGGAGTTGT GTTTTAGAGCTAGAAATAG
22	LIN28A	2	GTGGAAAGGACGAAACACCG CCCATCTCCAGTTGTGCGTG GTTTTAGAGCTAGAAATAG
23	LIN28A	3	GTGGAAAGGACGAAACACCG CGGGGTACTCAAGTCTTCTA GTTTTAGAGCTAGAAATAG
24	LIN28A	4	GTGGAAAGGACGAAACACCG TAATTATCTGCCCGGGGGGT GTTTTAGAGCTAGAAATAG
25	LIN28A	5	GTGGAAAGGACGAAACACCG TCTGATTGGCCAGCGCCGCC GTTTTAGAGCTAGAAATAG
26	NANOG	1	GTGGAAAGGACGAAACACCG TCCCAATTTACTGGGATTAC GTTTTAGAGCTAGAAATAG
27	NANOG	2	GTGGAAAGGACGAAACACCG TGATTTAAAAGTTGGAAACG GTTTTAGAGCTAGAAATAG
28	NANOG	3	GTGGAAAGGACGAAACACCG TCTAGTTCCCCACCTAGTCT GTTTTAGAGCTAGAAATAG
29	NANOG	4	GTGGAAAGGACGAAACACCG GATTAACTGAGAATTCACAA GTTTTAGAGCTAGAAATAG
30	NANOG	5	GTGGAAAGGACGAAACACCG CGCCAGGAGGGGTGGGTCTA GTTTTAGAGCTAGAAATAG
31	EEA	1	GTGGAAAGGACGAAACACCG CCCAGCACTTTGGG GTTTTAGAGCTAGAAATAG
32	EEA	2	GTGGAAAGGACGAAACACCG AATCCCAGCACTTT GTTTTAGAGCTAGAAATAG
33	EEA	3	GTGGAAAGGACGAAACACCG GCCTCCCAAAGTGC GTTTTAGAGCTAGAAATAG
34	EEA	7	GTGGAAAGGACGAAACACCG GCTACTTGGGAGGC GTTTTAGAGCTAGAAATAG
35	EEA	10	GTGGAAAGGACGAAACACCG GCCTCCCAAGTAGC GTTTTAGAGCTAGAAATAG
36	TdT	1	GTGGAAAGGACGAAACACCG GAGTTCGAGATCGA GTTTTAGAGCTAGAAATAG
37	TdT	2	GTGGAAAGGACGAAACACCG TTACGGGGCCGTCG GTTTTAGAGCTAGAAATAG
38	TdT	3	GTGGAAAGGACGAAACACCG AGCACGCCGTCGCG GTTTTAGAGCTAGAAATAG
39	TdT	4	GTGGAAAGGACGAAACACCG GGCCGCCCCTACGA GTTTTAGAGCTAGAAATAG
40	TdT	5	GTGGAAAGGACGAAACACCG CGTGATGAACTTCG GTTTTAGAGCTAGAAATAG
41	common ctrl	7	GTGGAAAGGACGAAACACCG ATTTTTAGTAGAGA GTTTTAGAGCTAGAAATAG
42	common ctrl	8	GTGGAAAGGACGAAACACCG TGGGAGGCTGAGGC GTTTTAGAGCTAGAAATAG
43	common ctrl	9	GTGGAAAGGACGAAACACCG AGTGCTGGGATTAC GTTTTAGAGCTAGAAATAG
44	common ctrl	10	GTGGAAAGGACGAAACACCG GTAGCTGGGATTAC GTTTTAGAGCTAGAAATAG
45	common ctrl	11	GTGGAAAGGACGAAACACCG CATGTTGGCCAGGC GTTTTAGAGCTAGAAATAG
46	ASCL1	1	GTGGAAAGGACGAAACACCG CGGGAGAAAGGAACGGGAGG GTTTTAGAGCTAGAAATAG
47	ASCL1	2	GTGGAAAGGACGAAACACCG AAGAACTTGAAGCAAAGCGC GTTTTAGAGCTAGAAATAG
48	ASCL1	3	GTGGAAAGGACGAAACACCG TCCAATTTCTAGGGTCACCG GTTTTAGAGCTAGAAATAG
49	ASCL1	4	GTGGAAAGGACGAAACACCG GTTGTGAGCCGTCCTGTAGG GTTTTAGAGCTAGAAATAG
51	NGN2	1	GTGGAAAGGACGAAACACCG GGCGGTGGCGGGGGAGGAGG GTTTTAGAGCTAGAAATAG
52	NGN2	2	GTGGAAAGGACGAAACACCG CAATGAAAAGAATAAGCCAG GTTTTAGAGCTAGAAATAG
53	NGN2	3	GTGGAAAGGACGAAACACCG GGGAAAGGCGGTGAAGAAAG GTTTTAGAGCTAGAAATAG
54	NGN2	4	GTGGAAAGGACGAAACACCG CGGAGCTGGCGAAGCCGCAG GTTTTAGAGCTAGAAATAG
56	GATA4	1	GTGGAAAGGACGAAACACCG ACCTCCAAGGAATCCGGGGC GTTTTAGAGCTAGAAATAG
57	GATA4	2	GTGGAAAGGACGAAACACCG CTCAACTCTCGATCTTGTGT GTTTTAGAGCTAGAAATAG
58	GATA4	3	GTGGAAAGGACGAAACACCG CAGCGAACCCAATCGACCTC GTTTTAGAGCTAGAAATAG
59	GATA4	4	GTGGAAAGGACGAAACACCG AATGCCCAAGTGCTACCGCC GTTTTAGAGCTAGAAATAG
60	GATA4	5	GTGGAAAGGACGAAACACCG CCTGTGGGAGTCACGTGCAA GTTTTAGAGCTAGAAATAG
61	FOXA2	1	GTGGAAAGGACGAAACACCG AGTGCCGAGCTGCCCCGAGG GTTTTAGAGCTAGAAATAG
62	FOXA2	2	GTGGAAAGGACGAAACACCG CGCGCGGCGCGGGGGCTAGT GTTTTAGAGCTAGAAATAG
63	FOXA2	3	GTGGAAAGGACGAAACACCG TGCGGCACTTGTCCGCTCCG GTTTTAGAGCTAGAAATAG
64	FOXA2	4	GTGGAAAGGACGAAACACCG TATAGCGCGGCGCGCTGGCG GTTTTAGAGCTAGAAATAG
65	FOXA2	5	GTGGAAAGGACGAAACACCG AAATGGGCTGCCCCGGGTCT GTTTTAGAGCTAGAAATAG
66	CDH1	1	GTGGAAAGGACGAAACACCG AGGGTCACCGCGTCTATGCG GTTTTAGAGCTAGAAATAG
67	CDH1	2	GTGGAAAGGACGAAACACCG CAGTGGAATCAGAACCGTGC GTTTTAGAGCTAGAAATAG
68	CDH1	3	GTGGAAAGGACGAAACACCG GTCTTAGTGAGCCACCGGCG GTTTTAGAGCTAGAAATAG
69	CDH1	4	GTGGAAAGGACGAAACACCG TCAGAAAGGGCTTTTACACT GTTTTAGAGCTAGAAATAG
70	CDH1	5	GTGGAAAGGACGAAACACCG GAGACAAGTCGGGGCGGACA GTTTTAGAGCTAGAAATAG
71	REX1	8	GTGGAAAGGACGAAACACCG TAGCAATACAGTCACATTAA GTTTTAGAGCTAGAAATAG
72	REX1	10	GTGGAAAGGACGAAACACCG CCGGGCAGAGAGTGAACGCG GTTTTAGAGCTAGAAATAG

Supplementary Table 2 | Addgene plasmids, related to dCas9 activator plasmid construction method

Addgene ID	Plasmid	Additional info
69536	pCXLE-dCas9VP192-T2A-EGFP	Balboa et al Stem Cell Reports. 2015 Sep 8;5(3):448-59. doi: 10.1016/j.stemcr.2015.08.001.
69535	pCXLE-dCas9VP192-T2A-EGFP-shP53	Balboa et al Stem Cell Reports. 2015 Sep 8;5(3):448-59. doi: 10.1016/j.stemcr.2015.08.001.
102885	PB-CAG-DDdCas9VP192-T2A-GFP-IRES-Neo	
102886	PB-CAG-DDdCas9VPH-T2A-GFP-IRES-Neo	
102887	PB-CAG-DDdCas9VPP300-T2A-GFP-IRES-Neo	
102888	PB-CAG-DDdCas9VPPH-T2A-GFP-IRES-Neo	
102889	PB-tight-DDdCas9VP192-T2A-GFP-IRES-Neo	
102890	PB-tight-DDdCas9VPH-T2A-GFP-IRES-Neo	
102891	PB-tight-DDdCas9VPP300-T2A-GFP-IRES-Neo	
102892	PB-tight-DDdCas9VPPH-T2A-GFP-IRES-Neo	
102893	PB-GG-OCT4-1-5-PGK-Puro	PiggyBac plasmid. Contains five guides targeting the human OCT4 promoter
102894	PB-GG-OMKSL-PGK-Puro	PiggyBac plasmid. Contains 3 guides for OCT4, 1 guide for MYC, 1 guide for KLF4, 2 guides for SOX2 and 3 guides for LIN28A promoters.
102895	pCXLE-dCas9VPH-T2A-GFP-shP53	
102896	pCXLE-dCas9VPP300-T2A-GFP-shP53	
102897	pCXLE-dCas9VPPH-T2A-GFP-shP53	
102898	GG-EBNA-EEA-5guides-PGK-Puro	Replicating episomal plasmid. Contains 5 guides targeting the EEA-motif.
102899	GG-EBNA-OMKSL-PP	Replicating episomal plasmid. Contains 3 guides for OCT4, 1 guide for MYC, 1 guide for KLF4, 2 guides for SOX2 and 3 guides for LIN28A promoters. Works better when combined with KM plasmid with extra guides for KLF4 and MYC.
102902	GG-EBNA-OSK2M2L1-PP	Replicating episomal plasmid. Contains 3 guides for OCT4, 2 guides for MYC, 2 guides for KLF4, 2 guides for SOX2 and 1 guides for LIN28A promoters.
102903	GG-EBNA-TdT-guide1-PGK-Puro	Replicating episomal plasmid. Control guide plasmid. Contains 1 guide targeting the TdTomato sequence.
102904	GG-EBNA-EEA-guide1-PGK-Puro	Replicating episomal plasmid. Contains guide nr.1 targeting the EEA-motif.
102906	pCXLE-dCas9GFP-shP53	
102907	PB-tight-DDdCas9GFP-IRES-Neo	
102908	PB-EEA-g1-PGK-Puro	PiggyBac plasmid. Contains guide nr.1 targeting the EEA-motif.
102909	PB-EEA-5g-OSK2M2L1-PGK-Puro	PiggyBac plasmid. Contains 5 guides targeting the EEA-motif, 3 guides for OCT4, 2 guides for MYC, 2 guides for KLF4, 2 guides for SOX2 and 1 guides for LIN28A promoters.

Supplementary Table 3 | PCR primers, related to qRT-PCR method

Gene	Reference	Forward	Reverse	Product size (bp)
CYCLOG	NM_004792	TCTTGTCAATGGCCAACAGAG	GCCCATCTAAATGAGGAGTTG	84
OCT4	NM_002701	TTGGGCTCGAGAAGGATGTG	TCCTCTCGTTGTGCATAGTCG	91
SOX2	NM_003106	GCCCTGCAGTACAACTCCAT	TGCCCTGCTGCGAGTAGGA	85
MYC	NM_002467	AGCGACTCTGAGGAGGAACA	CTCTGACCTTTTGCCAGGAG	87
NANOG	NM_024865.2	CTCAGCCTCCAGCAGATGC	TAGATTTCATTCTCTGGTTCTGG	94
REX1	NM_174900.3	CGTTTCGTGTGTCCCTTTCAA	CCTCTTGTTCATTCTTGTTCGT	106
LIN28	NM_024674	AGGAGACAGGTGCTACAACTG	TCTTGGGCTGGGGTGGCAG	74
KLF4	NM_004235.4	CCGCTCCATTACCAAG	CACGATCGTCTTCCCCTCTT	80
ASCL1	NM_004316.3	ACTCGTCGGACGAGGGCTCTTA	GCACTAAAGATGCAGGTTGTGCGA	153
NEUROG2	NM_024019.3	ATCCGAGCAGCACTAACACG	GCACAGGCCAAAGTCACAG	114
CDH1	NM_004360	ATGAGTGTCCCCCGGTATCT	GGTCAGTATCAGCCGCTTTC	91
FOXA2	NM_021784	AAGACCTACAGGCGCAGCT	CATCTTGTTGGGGCTCTGC	93
GATA4	NM_002052	GAGGAAGGAGCCAGCCTAGCAG	CGGGTCCCCCACTCGTCA	83
dCas9	-	AAACAGCAGATTCGCCTGGA	TCATCCGCTCGATGAAGCTC	113
mCherry	-	CCACTACGACGCTGAGGTCAA	TCGTTGTGGGAGGTGATGTCC	105
